# Supplementary material for: Spatial confinement induces oscillatory migration of epidermal keratinocytes and forms a three-compartment epithelial structure with epithelial–mesenchymal transition dynamics
Source: eLife. 2026 Nov 6;14:RP105192. doi: 10.7554/eLife.105192 (PMC13743076; doi:10.7554/eLife.105192)

Figure 4—figure supplement 1f

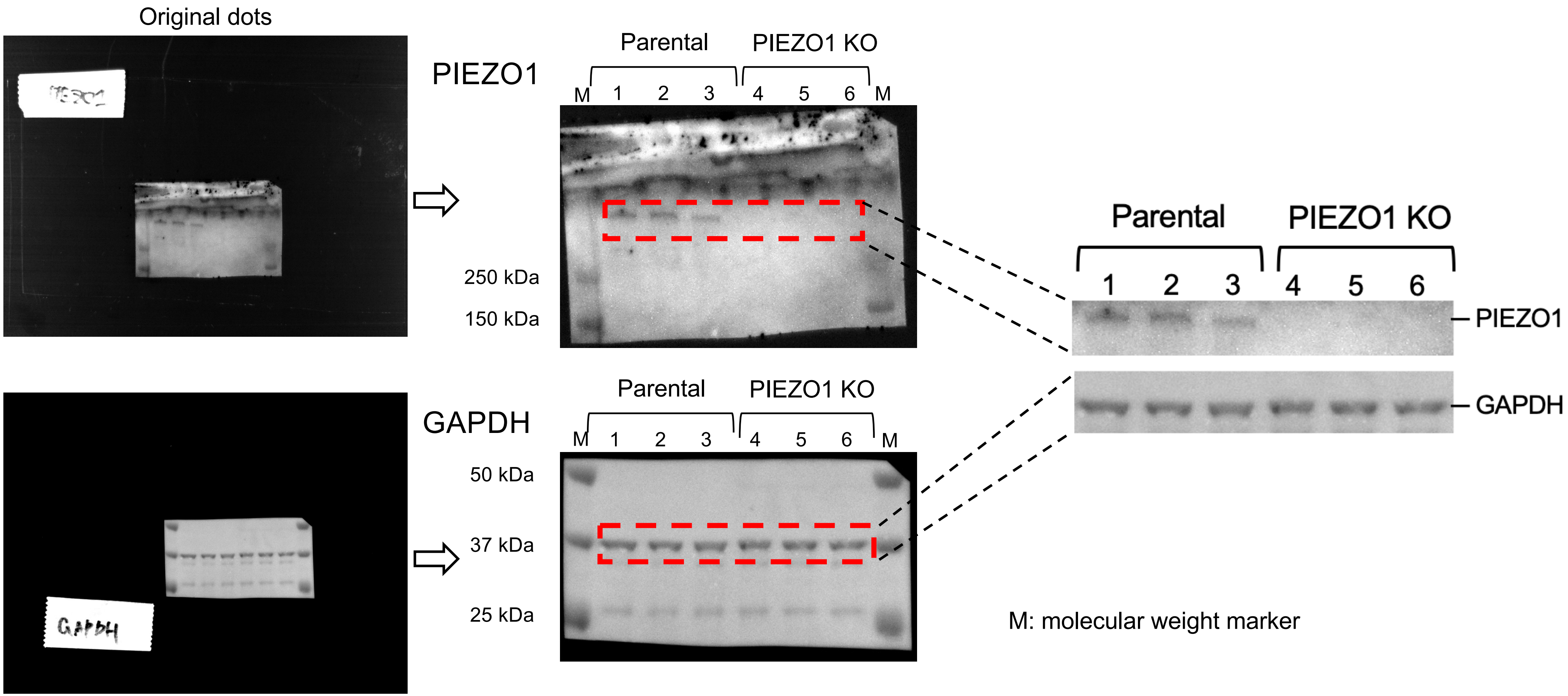

Supplement: Supplementary file 2 [file elife-105192-fig4-figsupp1-data2.zip › Figure 4ΓÇöfigure supplement 1f-source data 1.pdf]
