# Supplementary material for: Spatial confinement induces oscillatory migration of epidermal keratinocytes and forms a three-compartment epithelial structure with epithelial–mesenchymal transition dynamics
Source: eLife. 2026 Nov 6;14:RP105192. doi: 10.7554/eLife.105192 (PMC13743076; doi:10.7554/eLife.105192)
Supplement: Peer review history

# Editor report: eLife Assessment

**DOI:** 10.7554/eLife.105192.4.sa0

**Authors:** Beate Maria Lichtenberger

**Affiliations:** Medical University of Vienna Austria

**Keywords:** Compelling, Valuable

The authors established a novel three-dimensional culture system for stratified epithelia that allows epithelial cells to undergo epithelial-to-mesenchymal transition (EMT) and subsequent mesenchymal-to-epithelial transition (MET) while migrating through a membrane with 3.0-µm micropores, and, thus, provides a highly **valuable** tool to study EMT and possibly wound regeneration or metastasis. Furthermore, a set of experiments provides **compelling** data suggesting that TGF beta signaling and actin polymerization promote movement of epithelial cells into the pores, while Piezo1 and Keratin 6 prevent keratinocyte migration and EMT.

# Referee report: Reviewer #1 (Public review)

**DOI:** 10.7554/eLife.105192.4.sa1

[Editors' note: this version has been assessed by the Reviewing Editor without further input from the original reviewers. The authors have addressed the comments raised in the previous round of review.]

Summary:

The study describes the migration of epidermal keratinocytes through porous membranes and observed a unique size selection whereby only on 3-micron membrane are keratinocytes able to migrate and reform an intact epidermis. The authors propose that the model replicates three cell states of intact epidermis, EMT, and MET. They also show that this response depends on the actin cytoskeleton, keratin 6, and Piezo1, and the migration could be stimulated with TGFbeta ligands.

Strengths:

Strengths of the study include the establishment of a simple yet robust in vitro model that capture all three cell states, which could be useful for future investigation of wound healing or metastasis across a range of epithelia. There is also good quantitative characterisation of the pore size effects, providing some interesting observations about the physical regulation of keratinocyte migration, and the study provides some initial insights into the regulatory mechanisms. The images and presentation are clear.

# Referee report: Reviewer #2 (Public review)

**DOI:** 10.7554/eLife.105192.4.sa2

Summary:

The revised manuscript by Nohara et al. presents a novel 3D assay that allows for stratification of epithelia, active EMT through small pores, and active MET. They show that 3um pores allow for keratinocytes to sample the pore through filopodia and up regulate EMT genes to transverse the pores to the other side of the membrane where EMT genes are down regulated as the cells re-establish stratified epithelia. The TGFbeta pathway and actin polymerization promotes movement of cells into the pores and Piezo1 and KRT6 actively block this movement. This work provides a novel 3D assay that is likely to become a benchmark to analyze these processes using a more complex system than other current culture-specific EMT and MET assays.

Strengths:

The strengths of the manuscript include the foundational analysis of the pathways involved in establishing the 3-compartment epithelium. The authors have incorporated live imaging, drug studies, KO analysis, and RNA-sequencing to show the relevant pathways involved. The authors were responsive to the original round of reviews and present a much-improved study with greater details on molecular markers, quantification, and clarity.

# Referee report: Reviewer #3 (Public review)

**DOI:** 10.7554/eLife.105192.4.sa3

Summary:

The authors established an experimental system that reproduced three-dimensional triphasic epithelia, i.e., the original epithelium, its EMT, and MET. Keratinocytes (KCs), skin epithelial cells, placed on a microporous membrane migrated through 3.0-um or larger micropores. The 3.0-um-pored membrane induced an epithelial structure with three states: stratified KCs above the membrane, KCs showing EMT within the micropores, and a new stratified epithelium under the membrane. The membrane with larger micropores failed to maintain this triphasic epithelia. Live imaging revealed that KCs moved in a reciprocating manner, with actin-rich filopodia-like KC structures extending into and out of the 3.0-um micropores, while the cells migrated unidirectionally into larger micropores. KO of Piezo1 and keratin 6 increased KC entry to and exit from the 3.0-um micropores. Their results demonstrate that benign keratinocytes migrate through confined spaces in a reciprocating manner, which might help form triphasic epithelia, recapitulating wound healing processes.

Strengths:

Careful observation of the behaviour of keratinocytes on the different sized pores. CrispR-Cas9 gene editing to KO Piezo 1 and keratin 6 isoforms in HaCaT keratinocytes.

# Author comment: Author response

**DOI:** 10.7554/eLife.105192.4.sa4

**Authors:** Takuma Nohara, Sota Itamoto, Junichi Kumamoto, Yosuke Mai, Mayuna Shimano, Sora Kato, Hiroyuki Kitahata, Hideki Nakamura, Shota Takashima, Mika Watanabe, Masaharu Nagayama, Tsukasa Oikawa, Hideyuki Ujiie, Ken Natsuga

**Affiliations:** Hokkaido University Sapporo Japan; Hokkaido University Sapporo Japan; Hokkaido University Sapporo Japan; Hokkaido University Sapporo Japan; Department of Dermatology, Faculty of Medicine and Graduate School of Medicine, Hokkaido University, Sapporo, Japan Sapporo Japan; Department of Dermatology, Faculty of Medicine and Graduate School of Medicine, Hokkaido University, Sapporo, Japan Sapporo Japan; Chiba University Chiba Japan; Hokkaido University Sapporo Japan; Department of Dermatology, Faculty of Medicine and Graduate School of Medicine, Hokkaido University, Sapporo, Japan Sapporo Japan; Department of Dermatology, Faculty of Medicine and Graduate School of Medicine, Hokkaido University, Sapporo, Japan Sapporo Japan; Hokkaido University Sapporo Japan; Hokkaido University Sapporo Japan; Hokkaido University Sapporo Japan; Hokkaido University Sapporo Japan

The following is the authors’ response to the previous reviews.

Changes made independently of the reviewers' comments

In addition to the revisions described below, we have made the following three changes, none of which affects the conclusions of the paper.

(1) Shared control data (Figure 4c, d, l, m). The parental cells and the PIEZO1 KO and pan-KRT6 KO cell lines were assayed in parallel in the same experiments. We have therefore stated in the figure legend that the parental-cell data are shared between panels c, d and panels l, m.

(2) Number of DEGs (Figure 4h). We have corrected the number of genes upregulated in cells cultured on the 3.0-µm-pored membrane from 69 to 68.

(3) Yoda1 data (Figure 4e, f). Some values had been incorrectly transferred during data compilation. We have corrected these values and the corresponding graphs. The statistical results are unchanged.

We have also provided two new files: Source data 1 (numerical data underlying all graphs) and Source data 2 (uncropped western blot images).

**Public Reviews:**

**Reviewer #1 (Public review):**

Summary:

The study describes the migration of epidermal keratinocytes through porous membranes and observed a unique size selection whereby only on 3-micron membrane are keratinocytes able to migrate and reform an intact epidermis. The authors propose that the model replicates three cell states of intact epidermis, EMT, and MET. They also show that this response depends on the actin cytoskeleton, keratin 6, and Piezo1, and the migration could be stimulated with TGFbeta ligands.

Strengths:

Strengths of the study include the establishment of a simple yet robust in vitro model that capture all three cell states, which could be useful for future investigation of wound healing or metastasis across a range of epithelia. There is also good quantitative characterisation of the pore size effects, providing some interesting observations about the physical regulation of keratinocyte migration, and the study provides some initial insights into the regulatory mechanisms. The images and presentation are clear.

Weaknesses:

While there are some insights into the underlying mechanisms, there could be more detailed analysis of the key signalling pathways and functional relationships between actin, keratins, Piezo, and EMT genes. Although the extent of new biological insight is somewhat limited, the study still demonstrates the utility of the model, and these points may be important questions to address in future studies.

We agree with the reviewer that these are important questions. We will address the functional relationships among actin, keratins, PIEZO1, and EMT genes, and the underlying signaling pathways, in future studies.

**Reviewer #2 (Public review):**

Summary:

The revised manuscript by Nohara et al. presents a novel 3D assay that allows for stratification of epithelia, active EMT through small pores, and active MET. They show that 3um pores allow for keratinocytes to sample the pore through filopodia and up regulate EMT genes to transverse the pores to the other side of the membrane where EMT genes are down regulated as the cells re-establish stratified epithelia. The TGFbeta pathway and actin polymerization promotes movement of cells into the pores and Piezo1 and KRT6 actively block this movement. This work provides a novel 3D assay that is likely to become a benchmark to analyze these processes using a more complex system than other current culture-specific EMT and MET assays.

Strengths:

The strengths of the manuscript include the foundational analysis of the pathways involved in establishing the 3-compartment epithelium. The authors have incorporated live imaging, drug studies, KO analysis, and RNA-sequencing to show the relevant pathways involved. The authors were responsive to the original round of reviews and present a much-improved study with greater details on molecular markers, quantification, and clarity.

We thank the reviewer for the positive evaluation of our revised manuscript.

**Reviewer #3 (Public review):**

Summary:

The authors established an experimental system that reproduced three-dimensional triphasic epithelia, i.e., the original epithelium, its EMT, and MET. Keratinocytes (KCs), skin epithelial cells, placed on a microporous membrane migrated through 3.0-um or larger micropores. The 3.0-um-pored membrane induced an epithelial structure with three states: stratified KCs above the membrane, KCs showing EMT within the micropores, and a new stratified epithelium under the membrane. The membrane with larger micropores failed to maintain this triphasic epithelia. Live imaging revealed that KCs moved in a reciprocating manner, with actin-rich filopodia-like KC structures extending into and out of the 3.0-um micropores, while the cells migrated unidirectionally into larger micropores. KO of Piezo1 and keratin 6 increased KC entry to and exit from the 3.0-um micropores. Their results demonstrate that benign keratinocytes migrate through confined spaces in a reciprocating manner, which might help form triphasic epithelia, recapitulating wound healing processes.

Strengths:

Careful observation of the behaviour of keratinocytes on the different sized pores. CrispR-Cas9 gene editing to KO Piezo 1 and keratin 6 isoforms in HaCaT keratinocytes.

Weaknesses:

The authors have improved the observed weaknesses including better statistical analysis and more detail on replicates. They have also performed staining for laminin beta3 chain.

We thank the reviewer for these comments.

**Recommendations for the authors:**

**Reviewer #1 (Recommendations for the authors):**

The authors have significantly improved the manuscript in the recent revision. One minor point is to please make the RNA-seq analysis, including gene lists available in the supplementary information.

As suggested, we have added Figure 4—source data 1, which lists the differentially expressed genes (DEGs) identified by bulk RNA-seq of HaCaT keratinocytes cultured on 0.4-µm- or 3.0-µm-pored membranes.

**Reviewer #3 (Recommendations for the authors):**

I am not suggesting any further changes to the manuscript.

We thank the reviewer for evaluating our revised manuscript.
